# Supplementary material for: Meta-analysis reveals weak but pervasive plasticity in insect thermal limits
Source: Nat Commun. 2022 Sep 8;13:5292. doi: 10.1038/s41467-022-32953-2 (PMC9458737; doi:10.1038/s41467-022-32953-2)
Supplement: Supplementary file 1 — Supplementary information [file 41467_2022_32953_MOESM1_ESM.pdf]

**Supplementary Information**

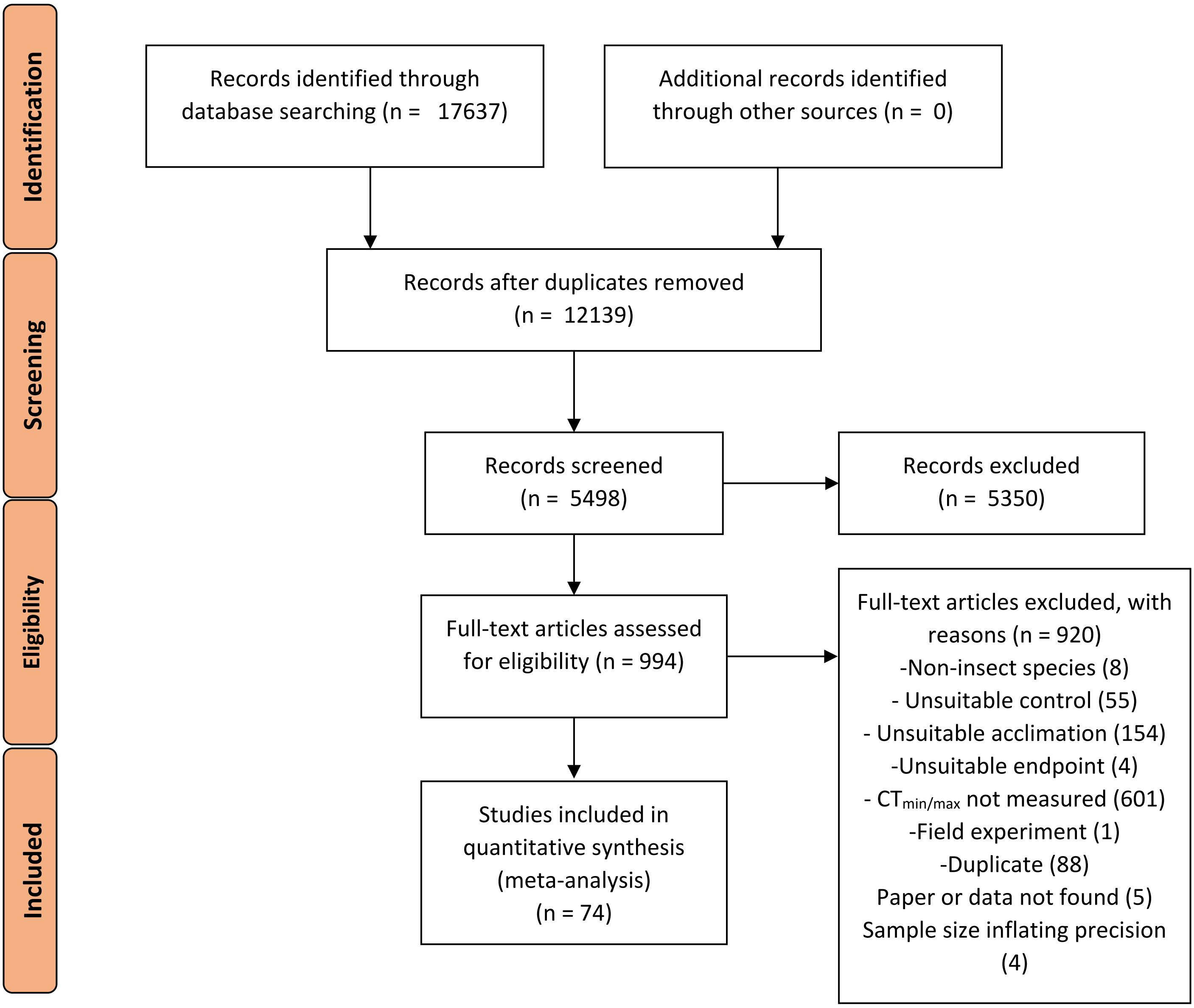


**Supplementary Figure 1** PRISMA exclusion procedure detailing how records were screened. First, duplicates were removed, then study titles were briefly screened to remove those clearly unsuitable e.g. non-insect species. Of the remaining articles, abstracts were assessed in detail and, finally, full methodology of selected studies was evaluated. Four articles were removed later in the analysis due studies measuring a very large number of insects at one time, meaning the sample size and therefore precision of the study was inflated. It was deemed that this many insects could not be assessed at once accurately.

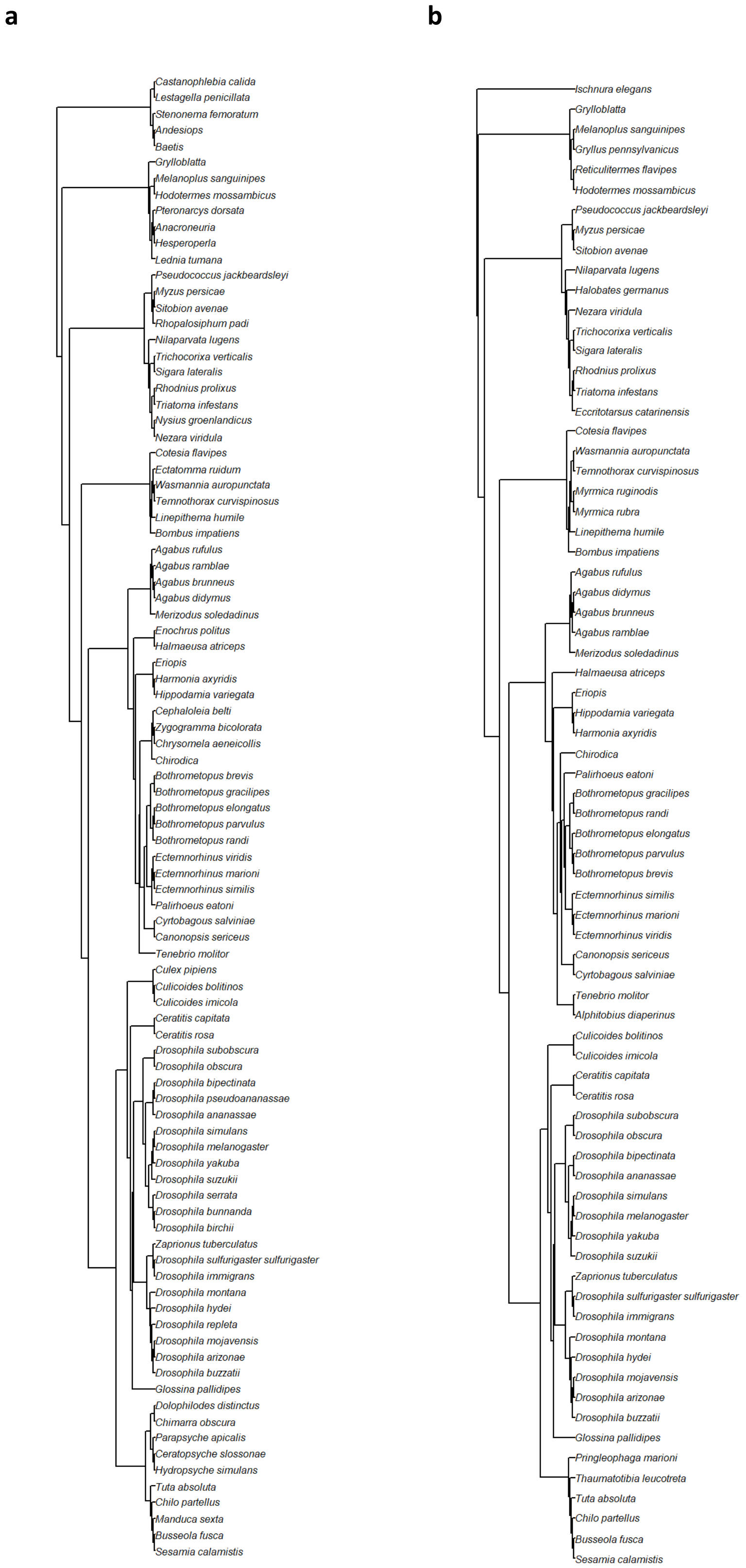


**Supplementary Figure 2** Phylogenetic tree used for (a) $CT_{max}$ and (b) $CT_{min}$ meta-analytic multi-level, random effects models. Phylogenetic trees were constructed using the Open Tree of Life and R packages ‘rotl’ and ‘ape’.

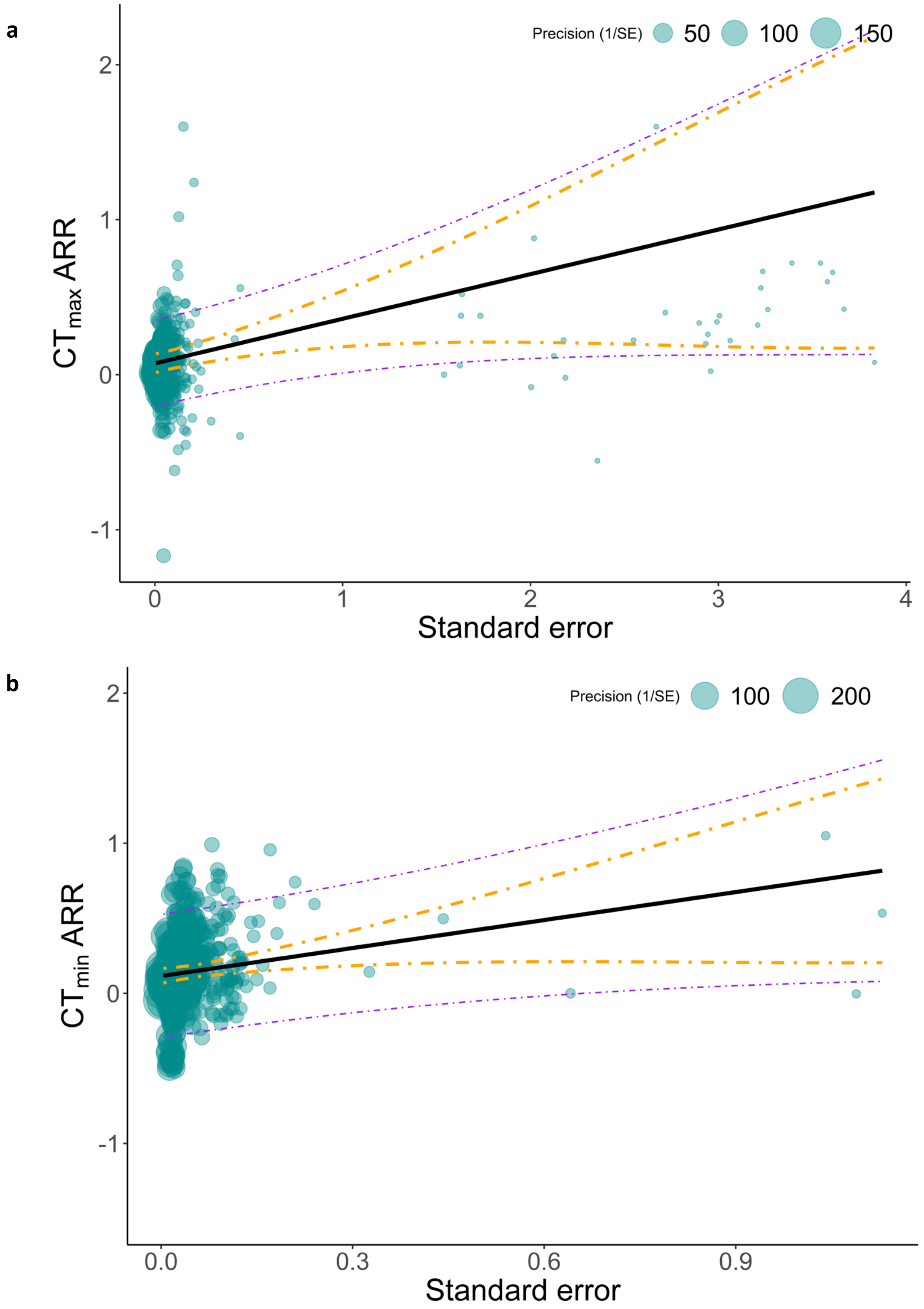


**Supplementary Figure 3** Relationship between (a) $CT_{max}$ and (b) $CT_{min}$ ARR (Acclimation Response Ratio) and standard error for Egger's regression test. A positive relationship shows positive publication bias. 95% confidence intervals are depicted by orange dotted lines, prediction intervals are purple dotted lines. The precision of the study (1/SE) is proportional to the size of each data point.

**Supplementary Table 1** List of heading names and descriptions in Supplementary Data 1

| Heading | Description |
|---|---|
| **rowid** | ID of row |
| **studyid** | ID of study |
| **popid** | ID of insect population |
| **ord** | Order |
| **fam** | Family |
| **gen** | Genus |
| **sp** | Species |
| **full_sp** | Genus + species. Some species have been changed to synonyms or to higher names in their taxonomy so they are recognised in the synthetic tree of life. |
| **habitat** | Aquatic or terrestrial |
| **source** | Laboratory or field source. Lab if one or more generations is under lab conditions. |
| **development** | Holometabolous or hemimetabolous |
| **acclim_stage** | Life stage at which acclimation took place |
| **ctm_stage** | Life stage at which CTL assay took place |
| **tsr** | Whether acclimation was within a life stage or between stages to investigate the temperature-size rule. |
| **sex** | Male, female, mixed or both/unknown |
| **lat** | Latitude in degrees |
| **stand_lat** | Latitude in degrees away from equator |
| **lat_bin** | Latitude in 10 degree bins |
| **mass** | Wet mass of insect (mg) extracted from original papers |
| **all_mass** | Wet mass of insect (mg) extracted from original papers and other sources |
| **acclim_temp** | Acclimation temperature (°C) |
| **cont_temp** | Control temperature (°C) |
| **acclim_dur** | Duration of acclimation period in hours. Data were only included if acclimation was stated in hours. If recorded as the length of a life stage, the cell is left blank. |
| **acclim_type** | Constant or fluctuating temperature during acclimation |
| **ctlimit** | $CT_{max}$ or $CT_{min}$ |
| **ramp_rate** | Rate of temp ramp (°C/min) |
| **groupID** | Treatments within a group use pairwise comparisons to calculate ARR (e.g., 10-12°C, 12-15°C, 15-20°C). |
| **treat** | CTL of treatment group |
| **treatSD** | Standard deviation of treatment group |
| **treatN** | Sample size of treatment group |
| **cont** | CTL of control group |
| **contSD** | Standard deviation of control group |
| **contN** | Sample size of control group |
| **simpctend** | End point at which critical thermal limit is measured, simplified into groupings |
| **data_type** | The source of the data collected e.g. table, graph, directly from author |
| **author** | First author of paper |
| **title** | Paper title |
| **journal** | Journal name |
| **year** | Year of publication |
| **webpage** | Link to paper webpage |
| **ARR** | Calculated Acclimation Response Ratio |
| **var** | Calculated variance |
| **precision** | Calculated precision (1/Standard Error) |
| **diff** | Difference between two acclimation temperatures used for ARR calculation |

**Supplementary Table 2** Intercept multi-level meta-analytic, random effects models for upper ($CT_{max}$) and lower ($CT_{min}$) critical thermal limits, to test whether ARR (Acclimation Response Ratio) is significantly different from zero. Significant results (95% CIs do not span zero) are highlighted in bold. CI.lb: lower bound of the 95% confidence interval; CI.ub: upper bound of the 95% confidence interval. $R^2_{marg.}$: $R^2$ marginal, the variance explained only by moderators. $R^2_{cond.}$: $R^2$ conditional, the variance explained by moderators and random effects.

| Limit | k | estimate | t | CI.lb | CI.ub | AICc | $I^2_{total}$ | $I^2_{study}$ | $I^2_{phylogeny}$ | $I^2_{species}$ | $I^2_{row}$ | $R^2_{marg.}$ | $R^2_{cond.}$ |
|---|---|---|---|---|---|---|---|---|---|---|---|---|---|
| **$CT_{max}$** | 803 | **0.091** | **2.98** | **0.030** | **0.153** | -797.2 | 0.972 | 0.151 | 0.153 | 0.177 | 0.491 | <0.001 | 0.495 |
| **$CT_{min}$** | 571 | **0.147** | **7.17** | **0.106** | **0.188** | -292.4 | 0.991 | 0.361 | <0.001 | <0.001 | 0.630 | <0.001 | 0.364 |

**Supplementary Table 3** Univariate multi-level meta-analytic, random effects models for upper critical thermal limit ($CT_{max}$) ARR (Acclimation Response Ratio) for all moderators. Results for intercept models are displayed. Results are highlighted in bold where 95% CIs do not overlap between groups or where regressions are significant for continuous variables. (rob.) is where a robust model was used because the residuals were not homogeneous. CI.lb: lower bound of the 95% confidence interval; CI.ub: upper bound of the 95% confidence interval. $R^2_{marg.}$: $R^2$ marginal, the variance explained only by moderators. $R^2_{cond.}$: $R^2$ conditional, the variance explained by moderators and random effects.

| Model | Comparison | k | estimate | t | CI.lb | CI.ub | AICc | $I^2_{total}$ | $I^2_{study}$ | $I^2_{phylogeny}$ | $I^2_{species}$ | $I^2_{row}$ | $R^2_{marg.}$ | $R^2_{cond.}$ |
|---|---|---|---|---|---|---|---|---|---|---|---|---|---|---|
| *~duration* | | 521 | <-0.001 | -0.05 | <-0.001 | <0.001 | -588.3 | 0.963 | 0.351 | 0.345 | 0.267 | <0.001 | <0.001 | 0.642 |
| | Intercept | - | 0.077 | 2.27 | 0.009 | 0.145 | - | - | - | - | - | - | - | - |
| *~ramp rate* | | 803 | 0.020 | 0.45 | -0.067 | 0.106 | -794.8 | 0.973 | 0.162 | 0.479 | 0.160 | 0.171 | 0.001 | 0.508 |
| | Intercept | - | 0.085 | 2.44 | 0.015 | 0.155 | - | - | - | - | - | - | - | - |
| *~mass* (rob.) | | 614 | <0.001 | 0.94 | -0.001 | 0.003 | -689.3 | 0.976 | 0.032 | 0.443 | 0.313 | 0.188 | 0.047 | 0.567 |
| | Intercept | - | 0.142 | 2.74 | 0.024 | 0.153 | - | - | - | - | - | - | - | - |
| ***~acclimation stage*** | **Early life** | 803 | **0.105** | **3.98** | **0.053** | **0.156** | -791.4 | 0.970 | 0.157 | 0.530 | 0.094 | 0.189 | 0.013 | 0.461 |
| | **Adulthood** | - | **0.069** | **2.63** | **0.017** | **0.120** | - | - | - | - | - | - | - | - |
| | Whole life | - | 0.076 | 2.63 | 0.019 | 0.132 | - | - | - | - | - | - | - | - |
| | Several generations | - | 0.080 | 1.85 | -0.005 | 0.164 | - | - | - | - | - | - | - | - |

| | | | | | | | | | | | | | | |
|---|---|---|---|---|---|---|---|---|---|---|---|---|---|---|
| *~source* | Laboratory | 803 | 0.105 | 2.87 | 0.032 | 0.177 | -795.4 | 0.973 | 0.162 | 0.175 | 0.163 | 0.472 | 0.004 | 0.517 |
| | Field | - | 0.083 | 2.34 | 0.013 | 0.154 | - | - | - | - | - | - | - | - |
| *~habitat* (rob.) | Aquatic | 803 | 0.090 | 2.01 | <0.001 | 0.180 | -795.0 | 0.973 | 0.160 | 0.166 | 0.167 | 0.479 | <0.001 | 0.507 |
| | Terrestrial | - | 0.093 | 2.74 | 0.025 | 0.161 | - | - | - | - | - | - | - | - |
| ***~end point*** | Death | 803 | 0.071 | 0.62 | -0.160 | 0.302 | -794.3 | 0.974 | 0.205 | 0.126 | 0.198 | 0.446 | 0.076 | 0.577 |
| | **Activity** | - | **0.085** | **2.01** | **0.002** | **0.167** | - | - | - | - | - | - | - | - |
| | **Clinging** | - | **0.072** | **1.19** | **-0.047** | **0.191** | - | - | - | - | - | - | - | - |
| | **Righting** | - | **0.212** | **3.97** | **0.105** | **0.318** | - | - | - | - | - | - | - | - |
| | **Stimulus response** | - | **0.050** | **1.15** | **-0.035** | **0.136** | - | - | - | - | - | - | - | - |
| | **Motor response** | - | **0.078** | **1.92** | **-0.002** | **0.158** | - | - | - | - | - | - | - | - |
| *~latitude* | | 701 | -0.001 | -1.20 | -0.002 | <0.001 | -607.8 | 0.972 | 0.294 | 0.071 | 0.148 | 0.459 | 0.004 | 0.530 |
| | Intercept | - | 0.115 | 2.81 | 0.033 | 0.198 | - | - | - | - | - | - | - | - |
| *~latitude* (field- | | 187 | -0.001 | -0.72 | -0.005 | 0.003 | 12.4 | 0.944 | 0.380 | 0.032 | <0.001 | 0.532 | 0.010 | 0.441 |
| caught subset) | Intercept | - | 0.106 | 1.38 | -0.053 | 0.265 | - | - | - | - | - | - | - | - |
| *~sex* | Female | 803 | 0.057 | 1.55 | -0.016 | 0.130 | -794.0 | 0.972 | 0.158 | 0.143 | 0.191 | 0.480 | 0.022 | 0.517 |
| | Male | - | 0.093 | 2.32 | 0.014 | 0.171 | - | - | - | - | - | - | - | - |
| | Both | - | 0.087 | 2.20 | 0.010 | 0.164 | - | - | - | - | - | - | - | - |
| | Unknown | - | 0.108 | 3.24 | 0.043 | 0.173 | - | - | - | - | - | - | - | - |
| ***~development*** | **Holometabolous** | 803 | **0.045** | **1.48** | **-0.015** | **0.105** | -799.2 | 0.970 | 0.166 | 0.080 | 0.201 | 0.523 | 0.058 | 0.492 |
| | **Hemimetabolous** | - | **0.135** | **3.95** | **0.067** | **0.203** | - | - | - | - | - | - | - | - |
| *~development* | Holometabolous | 591 | 0.034 | 1.28 | -0.020 | 0.087 | -777.4 | 0.960 | 0.006 | 0.097 | 0.093 | 0.763 | 0.025 | 0.224 |
| (juvenile subset) | Hemimetabolous | - | 0.092 | 2.54 | 0.019 | 0.165 | - | - | - | - | - | - | - | - |
| Temperature-size | Between | 803 | 0.091 | 2.66 | 0.024 | 0.158 | 793.9 | 0.972 | 0.159 | 0.157 | 0.168 | 0.488 | <0.001 | 0.498 |
| rule | Within | - | 0.092 | 2.95 | 0.031 | 0.153 | - | - | - | - | - | - | - | - |

**Supplementary Table 4** Univariate multi-level meta-analytic, random effects models for lower critical thermal limit ($CT_{min}$) ARR (Acclimation Response Ratio) for all moderators. Results for intercept models are displayed. Results are highlighted in bold where 95% CIs do not overlap between groups or where regressions are significant for continuous variables. (rob.) is where a robust model was used because the residuals were not homogeneous. CI.lb: lower bound of the 95% confidence interval; CI.ub: upper bound of the 95% confidence interval. $R^2_{marg.}$: $R^2$ marginal, the variance explained only by moderators. $R^2_{cond.}$: $R^2$ conditional, the variance explained by moderators and random effects.

| Model | Comparison | k | estimate | t | CI.lb | CI.ub | AICc | $I^2_{total}$ | $I^2_{study}$ | $I^2_{phylogeny}$ | $I^2_{species}$ | $I^2_{row}$ | $R^2_{marg.}$ | $R^2_{cond.}$ |
|---|---|---|---|---|---|---|---|---|---|---|---|---|---|---|
| ~*duration* (rob.) | | 375 | <0.001 | 0.64 | <-0.001 | <0.001 | -291.8 | 0.989 | 0.232 | 0.016 | <0.001 | 0.741 | 0.018 | 0.264 |
| | Intercept | - | 0.098 | 5.52 | 0.062 | 0.133 | - | - | - | - | - | - | - | - |
| ~*ramp rate* (rob.) | | 571 | 0.017 | 0.31 | -0.091 | 0.125 | -290.7 | 0.992 | 0.367 | <0.001 | <0.001 | 0.625 | <0.001 | 0.370 |
| | Intercept | - | 0.142 | 5.41 | 0.089 | 0.194 | - | - | - | - | - | - | - | - |
| ~*mass* | | 464 | <-0.001 | -0.63 | -0.001 | <0.001 | -205.8 | 0.992 | 0.992 | 0.399 | <0.001 | <0.001 | 0.594 | 0.002 |
| | Intercept | - | 0.151 | 5.72 | 0.098 | 0.204 | - | - | - | - | - | - | - | - |
| ***~acclimation stage*** | **Early life** | 571 | **0.196** | **6.36** | **0.136** | **0.257** | -289.7 | 0.992 | 0.372 | <0.001 | <0.001 | 0.619 | 0.016 | 0.385 |
| | **Adulthood** | - | **0.129** | **5.43** | **0.082** | **0.176** | - | - | - | - | - | - | - | - |
| | Whole life | - | 0.128 | 3.27 | 0.051 | 0.204 | - | - | - | - | - | - | - | - |
| | Several generations | - | 0.164 | 2.47 | 0.034 | 0.295 | - | - | - | - | - | - | - | - |
| ***~source*** **(rob.)** | **Laboratory** | 571 | **0.163** | **8.83** | **0.126** | **0.200** | -292.3 | 0.991 | 0.348 | <0.001 | <0.001 | 0.643 | 0.012 | 0.359 |
| | **Field** | - | **0.112** | **6.21** | **0.076** | **0.147** | - | - | - | - | - | - | - | - |
| ~*habitat* | Aquatic | 571 | 0.038 | 0.43 | -0.139 | 0.215 | -291.9 | 0.991 | 0.358 | <0.001 | <0.001 | 0.633 | 0.010 | 0.368 |
| | Terrestrial | - | 0.153 | 7.31 | 0.111 | 0.195 | - | - | - | - | - | - | - | - |
| ***~end point*** **(rob.)** | **Death** | 571 | **0.052** | **1.59** | **-0.013** | **0.117** | -283.4 | 0.992 | 0.376 | <0.001 | <0.001 | 0.616 | 0.023 | 0.393 |
| | Activity | - | 0.152 | 2.52 | 0.032 | 0.272 | - | - | - | - | - | - | - | - |
| | **Clinging** | - | **0.150** | **4.65** | **0.085** | **0.214** | - | - | - | - | - | - | - | - |
| | **Natural position** | - | **0.038** | **2.14** | **0.003** | **0.073** | - | - | - | - | - | - | - | - |
| | **Righting** | - | **0.168** | **4.11** | **0.086** | **0.249** | - | - | - | - | - | - | - | - |

| | | | | | | | | | | | | | | |
|---|---|---|---|---|---|---|---|---|---|---|---|---|---|---|
| | **Stimulus response** | - | **0.146** | **4.16** | **0.076** | **0.216** | - | - | - | - | - | - | - | - |
| | **Motor response** | - | **0.172** | **4.06** | **0.087** | **0.256** | - | - | - | - | - | - | - | - |
| ~*latitude* | | 471 | -0.001 | -0.97 | -0.002 | 0.001 | -207.1 | 0.990 | 0.369 | <0.001 | <0.001 | 0.622 | 0.004 | 0.375 |
| | Intercept | - | 0.169 | 4.26 | 0.089 | 0.250 | - | - | - | - | - | - | - | - |
| ~*latitude* (field | | 144 | -0.001 | -1.64 | -0.002 | <0.001 | -188.7 | 0.946 | <0.001 | 0.048 | <0.001 | 0.898 | 0.019 | 0.069 |
| subset; rob.) | Intercept | - | 0.127 | 4.02 | 0.062 | 0.191 | - | - | - | - | - | - | - | - |
| ~*sex* | Female | 571 | 0.186 | 5.80 | 0.123 | 0.249 | -289.5 | 0.991 | 0.359 | <0.001 | <0.001 | 0.632 | 0.024 | 0.377 |
| | Male | - | 0.158 | 4.26 | 0.085 | 0.231 | - | - | - | - | - | - | - | - |
| | Both | - | 0.164 | 5.19 | 0.102 | 0.226 | - | - | - | - | - | - | - | - |
| | Unknown | - | 0.096 | 2.75 | 0.028 | 0.165 | - | - | - | - | - | - | - | - |
| ~*development* | Holometabolous | 571 | 0.138 | 5.83 | 0.090 | 0.186 | -291.1 | 0.992 | 0.3633 | <0.001 | <0.001 | 0.629 | 0.004 | 0.368 |
| | Hemimetabolous | - | 0.174 | 4.22 | 0.091 | 0.257 | - | - | - | - | - | - | - | - |
| ~*development* (juvenile subset) | Holometabolous | 473 | 0.126 | 4.51 | 0.070 | 0.183 | -263.4 | 0.991 | 0.429 | <0.001 | <0.001 | 0.562 | 0.013 | 0.440 |
| | Hemimetabolous | - | 0.189 | 3.60 | 0.083 | 0.296 | - | - | - | - | - | - | - | - |
| Temperature-size rule | Between | 571 | 0.114 | 2.88 | 0.036 | 0.191 | -291.1 | 0.992 | 0.395 | <0.001 | <0.001 | 0.597 | 0.007 | 0.402 |
| | Within | - | 0.152 | 6.87 | 0.108 | 0.195 | - | - | - | - | - | - | - | - |

**Supplementary Table 5** Multivariate multi-level meta-analytic, random effects models for upper critical thermal limit ($CT_{max}$) ARR (Acclimation Response Ratio). Top four best models are shown, calculated using the 'dredge' from the MuMIn package. Estimate: difference in ARR compared to the reference group or the coefficient for regressions. Reference groups are indicated in subscript. Results are highlighted in bold where 95% CIs do not overlap between groups or where regressions are significant for continuous variables. CI.lb: lower bound of the 95% confidence interval; CI.ub: upper bound of the 95% confidence interval. $R^2$ marg.: $R^2$ marginal, the variance explained only by moderators. $R^2$ cond.: $R^2$ conditional, the variance explained by moderators and random effects.

| Rank | Model | Comparison | k | estimate | t | CI.lb | CI.ub | AICc | $I^2_{total}$ | $I^2_{study}$ | $I^2_{phylogeny}$ | $I^2_{species}$ | $I^2_{row}$ | $R^2_{marg.}$ | $R^2_{cond.}$ |
|---|---|---|---|---|---|---|---|---|---|---|---|---|---|---|---|
| 1 | ***~development*** $_{hemimetabolous}$ | **Holometabolous** | 803 | **-0.090** | **-2.12** | **-0.175** | **-0.006** | -799.2 | 0.970 | 0.165 | 0.077 | 0.204 | 0.524 | 0.059 | 0.491 |
| 2 | ***~development*** $_{hemimetabolous}$ + ***acclimation stage*** $_{adulthood}$ | **Holometabolous** | 803 | **-0.073** | **-2.02** | **-0.145** | **-0.001** | -792.5 | 0.969 | 0.165 | 0.037 | 0.214 | 0.551 | 0.064 | 0.467 |
| | | **Early life** | - | **0.034** | **2.29** | **0.005** | **0.064** | - | - | - | - | - | - | - | - |
| | | Whole life | - | 0.007 | 0.43 | -0.024 | 0.038 | - | - | - | - | - | - | - | - |
| | | Several generations | - | 0.009 | 0.24 | -0.062 | 0.080 | - | - | - | - | - | - | - | - |
| 3 | ***~development*** $_{hemimetabolous}$ + *source* $_{field\text{-}caught}$ | **Holometabolous** | 803 | **-0.095** | **-2.12** | **-0.185** | **-0.006** | -797.5 | 0.971 | 0.176 | 0.087 | 0.197 | 0.510 | 0.061 | 0.507 |
| | | Laboratory | | 0.023 | 0.82 | -0.033 | 0.080 | | | | | | | | |
| 4 | ***~development*** $_{hemimetabolous}$ + ***acclimation stage*** $_{adulthood}$ + *source* $_{field\text{-}caught}$ | **Holometabolous** | 803 | **-0.078** | **-2.04** | **-0.154** | **-0.002** | -790.4 | 0.969 | 0.178 | 0.044 | 0.209 | 0.538 | 0.065 | 0.481 |
| | | **Early life** | - | **0.033** | **2.21** | **0.004** | **0.063** | - | - | - | - | - | - | - | - |
| | | Whole life | - | 0.005 | 0.32 | -0.027 | 0.037 | - | - | - | - | - | - | - | - |
| | | Several generations | - | 0.007 | 0.18 | -0.065 | 0.078 | - | - | - | - | - | - | - | - |
| | | Laboratory | - | 0.019 | 0.69 | -0.036 | 0.074 | - | - | - | - | - | - | - | - |

**Supplementary Table 6** Multivariate multi-level meta-analytic, random effects models for lower critical thermal limit ($CT_{min}$) ARR (Acclimation Response Ratio). Top four best models are shown, calculated using the 'dredge' from the MuMIn package. Estimate: difference in ARR compared to the reference group or the coefficient for regressions. Reference groups are indicated in subscript. Results are highlighted in bold where 95% CIs do not overlap between groups or where regressions are significant for continuous variables. CI.lb: lower bound of the 95% confidence interval; CI.ub: upper bound of the 95% confidence interval. $R^2$ marg.: $R^2$ marginal, the variance explained only by moderators. $R^2$ cond.: $R^2$ conditional, the variance explained by moderators and random effects.

| Rank | Model | Comparison | k | estimate | t | CI.lb | CI.ub | AICc | $I^2_{total}$ | $I^2_{study}$ | $I^2_{phylogeny}$ | $I^2_{species}$ | $I^2_{row}$ | $R^2_{marg.}$ | $R^2_{cond.}$ |
|---|---|---|---|---|---|---|---|---|---|---|---|---|---|---|---|
| 1 | ***~source** $_{field\text{-}caught}$* **(rob.)** | **Laboratory** | 571 | 0.052 | **2.24** | **0.006** | **0.098** | -292.3 | 0.991 | 0.348 | <0.001 | <0.001 | 0.643 | 0.012 | 0.359 |
| 2 | *Intercept model* | - | 571 | **0.147** | **7.17** | **0.106** | **0.188** | -292.4 | 0.991 | 0.361 | <0.001 | <0.001 | 0.630 | <0.001 | 0.364 |
| 3 | ***~sex** $_{female}$ + acclimation stage $_{adulthood}$* (rob.) | Male | 571 | -0.024 | -0.68 | -0.048 | <0.001 | -289.1 | 0.992 | 0.375 | <0.001 | <0.001 | 0.616 | 0.055 | 0.413 |
| | | Mixed | - | -0.024 | -0.61 | -0.098 | 0.050 | - | - | - | - | - | - | - | - |
| | | **Unknown** | **-** | **0.124** | **-2.54** | **-0.211** | **-0.036** | - | - | - | - | - | - | - | - |
| | | Early life | - | 0.084 | 2.52 | 0.027 | 0.141 | - | - | - | - | - | - | - | - |
| | | Whole life | - | -0.014 | -0.33 | -0.195 | 0.168 | - | - | - | - | - | - | - | - |
| | | Several generations | - | 0.022 | 0.32 | -0.144 | 0.188 | - | - | - | - | - | - | - | - |
| 4 | *~habitat$_{aquatic}$* | Terrestrial | 571 | 0.115 | 1.27 | -0.067 | 0.297 | -291.9 | 0.991 | 0.358 | <0.001 | <0.001 | 0.633 | 0.010 | 0.368 |

**Supplementary Table 7** Conditional average best moderators from multivariate multi-level meta-analytic, random effects models for upper thermal limit ($CT_{max}$) ARR (Acclimation Response Ratio), ranked by AICc. Estimate: difference in ARR compared to the reference group or the coefficient for regressions. Significant results (95% CIs do not overlap between groups or significant regressions for continuous variables) are highlighted in bold. Reference groups are indicated in subscript. SE: standard error. CI.lb: lower bound of the 95% confidence interval; CI.ub: upper bound of the 95% confidence interval.

| Moderator | estimate | SE | z | CI.lb | CI.ub |
|---|---|---|---|---|---|
| **Development: holometabolous** $_{\text{hemimetabolous}}$ | **-0.080** | 0.030 | **2.65** | **-0.139** | **-0.020** |
| **Acclimation stage: Early life** $_{\text{adulthood}}$ | **0.036** | 0.015 | **2.46** | **0.007** | **0.065** |
| Acclimation stage: Several generations $_{\text{adulthood}}$ | 0.008 | 0.036 | 0.22 | -0.063 | 0.079 |
| Acclimation stage: Whole life $_{\text{adulthood}}$ | 0.007 | 0.016 | 0.46 | -0.024 | 0.038 |
| Source: Laboratory $_{\text{field-caught}}$ | 0.019 | 0.026 | 0.72 | -0.032 | 0.070 |
| Ramp rate | 0.013 | 0.041 | 0.31 | -0.068 | 0.093 |

**Supplementary Table 8** Full average best moderators from multivariate multi-level meta-analytic, random effects models for upper thermal limit ($CT_{max}$) ARR (Acclimation Response Ratio), ranked by AICc. Estimate: difference in ARR compared to the reference group or the coefficient for regressions. Significant results (95% CIs do not overlap between groups or significant regressions for continuous variables) are highlighted in bold. Reference groups are indicated in subscript. SE: standard error. CI.lb: lower bound of the 95% confidence interval; CI.ub: upper bound of the 95% confidence interval.

| Moderator | estimate | SE | z | CI.lb | CI.ub |
|---|---|---|---|---|---|
| **Development: holometabolous** $_{\text{hemimetabolous}}$ | **-0.080** | 0.030 | **2.65** | **-0.139** | **-0.021** |
| Acclimation stage: Early life $_{\text{adulthood}}$ | 0.015 | 0.020 | 0.74 | -0.025 | 0.055 |
| Acclimation stage: Several generations $_{\text{adulthood}}$ | 0.003 | 0.024 | 0.14 | -0.043 | 0.049 |
| Acclimation stage: Whole life $_{\text{adulthood}}$ | 0.003 | 0.011 | 0.28 | -0.018 | 0.024 |
| Source: Laboratory $_{\text{field-caught}}$ | 0.005 | 0.016 | 0.33 | -0.026 | 0.037 |
| Ramp rate | 0.002 | 0.015 | 0.10 | -0.027 | 0.030 |

**Supplementary Table 9** Conditional average best moderators from multivariate multi-level meta-analytic, random effects models for lower thermal limit ($CT_{min}$) ARR (Acclimation Response Ratio), ranked by AICc. Estimate: difference in ARR compared to the reference group or the coefficient for regressions. Significant results (95% CIs do not overlap between groups or significant regressions for continuous variables) are highlighted in bold. Reference groups are indicated in subscript. SE: standard error. CI.lb: lower bound of the 95% confidence interval; CI.ub: upper bound of the 95% confidence interval.

| Moderator | estimate | SE | z | CI.lb | CI.ub |
|---|---|---|---|---|---|
| Source: Laboratory $_{\text{field-caught}}$ | 0.049 | 0.036 | 1.38 | -0.021 | 0.119 |
| **Acclimation stage: Early life $_{\text{adulthood}}$** | **0.079** | 0.034 | **2.34** | **0.0128** | **0.144** |
| Acclimation stage: Several generations $_{\text{adulthood}}$ | 0.030 | 0.067 | 0.45 | -0.102 | 0.162 |
| Acclimation stage: Whole life $_{\text{adulthood}}$ | -0.005 | 0.040 | 0.11 | -0.083 | 0.074 |
| Sex: Male $_{\text{female}}$ | -0.027 | 0.035 | 0.77 | -0.096 | 0.042 |
| Sex: Mixed $_{\text{female}}$ | -0.025 | 0.039 | 0.64 | -0.100 | 0.051 |
| **Sex: Unknown $_{\text{female}}$** | **-0.121** | 0.047 | **2.56** | **-0.213** | **-0.028** |
| Habitat: Terrestrial $_{\text{aquatic}}$ | 0.114 | 0.093 | 1.23 | -0.068 | 0.2961 |
| Development: Holometabolous $_{\text{hemimetabolous}}$ | -0.050 | 0.048 | 1.06 | -0.143 | 0.043 |
| Ramp rate | 0.021 | 0.069 | 0.30 | -0.114 | 0.155 |

**Supplementary Table 10** Full average best moderators from multivariate multi-level meta-analytic, random effects models for lower thermal limit ($CT_{min}$) ARR (Acclimation Response Ratio), ranked by AICc. Estimate: difference in ARR compared to the reference group or the coefficient for regressions. Significant results (95% CIs do not overlap between groups or significant regressions for continuous variables) are highlighted in bold. Reference groups are indicated in subscript. SE: standard error. CI.lb: lower bound of the 95% confidence interval; CI.ub: upper bound of the 95% confidence interval.

| **Moderator** | **estimate** | **SE** | **z** | **CI.lb** | **CI.ub** |
|---|---|---|---|---|---|
| Source: Laboratory $_{\text{field-caught}}$ | 0.023 | 0.035 | 0.67 | 0.091 | -0.045 |
| Acclimation stage: Early life $_{\text{adulthood}}$ | 0.042 | 0.046 | 0.90 | 0.132 | -0.049 |
| Acclimation stage: Several generations $_{\text{adulthood}}$ | 0.016 | 0.051 | 0.31 | 0.116 | -0.084 |
| Acclimation stage: Whole life $_{\text{adulthood}}$ | -0.002 | 0.029 | 0.08 | 0.055 | -0.060 |
| Sex: Male $_{\text{female}}$ | -0.009 | 0.024 | 0.39 | 0.039 | -0.057 |
| Sex: Mixed $_{\text{female}}$ | -0.009 | 0.026 | 0.33 | 0.042 | -0.059 |
| Sex: Unknown $_{\text{female}}$ | -0.042 | 0.064 | 0.66 | 0.083 | -0.167 |
| Habitat: Terrestrial $_{\text{aquatic}}$ | 0.046 | 0.081 | 0.57 | 0.205 | -0.113 |
| Development: Holometabolous $_{\text{hemimetabolous}}$ | -0.014 | 0.034 | 0.42 | 0.053 | -0.082 |
| Ramp rate | 0.001 | 0.013 | 0.05 | 0.026 | -0.024 |

**Sensitivity analyses**

**Supplementary Table 11** Leave-one-out sensitivity analysis for multi-level meta-analytic, random effects models of upper thermal limit ($CT_{max}$) ARR (Acclimation Response Ratio). Individual studies, species or families were removed iteratively to check for outliers. SE: standard error. CI.lb: lower bound of the 95% confidence interval; CI.ub: upper bound of the 95% confidence interval.

| **Moderator** | **estimate** | **z** | **SE** | **CI.lb** | **CI.ub** |
|---|---|---|---|---|---|
| Study | 0.091 | 2.96 | 0.031 | 0.030 | 0.153 |
| Species | 0.091 | 2.97 | 0.031 | 0.030 | 0.153 |
| Family | 0.091 | 2.96 | 0.031 | 0.030 | 0.153 |

**Supplementary Table 12** Leave-one-out sensitivity analysis for multi-level meta-analytic, random effects models of lower thermal limit ($CT_{min}$) ARR (Acclimation Response Ratio). Individual studies, species or families were removed iteratively to check for outliers. SE: standard error. CI.lb: lower bound of the 95% confidence interval; CI.ub: upper bound of the 95% confidence interval.

| **Moderator** | **estimate** | **z** | **SE** | **CI.lb** | **CI.ub** |
|---|---|---|---|---|---|
| Study | 0.147 | 7.08 | 0.021 | 0.105 | 0.189 |
| Species | 0.147 | 7.11 | 0.021 | 0.105 | 0.189 |
| Family | 0.147 | 7.04 | 0.021 | 0.105 | 0.189 |

**Supplementary Table 13** Intercept multi-level meta-analytic, random effects models for upper and lower thermal limits, $CT_{max}$ and $CT_{min}$ respectively, without fluctuating data. CI.lb: lower bound of the 95% confidence interval; CI.ub: upper bound of the 95% confidence interval. $R^2_{marg.}$: $R^2$ marginal, the variance explained only by moderators. $R^2_{cond.}$: $R^2$ conditional, the variance explained by moderators and random effects. Significant results (95% CIs do not span 0) are highlighted in bold.

| **Limit** | **k** | **estimate** | **t** | **CI.lb** | **CI.ub** | **AICc** | **$I^2_{total}$** | **$I^2_{study}$** | **$I^2_{phylogeny}$** | **$I^2_{species}$** | **$I^2_{row}$** | **$R^2_{marg.}$** | **$R^2_{cond.}$** |
|---|---|---|---|---|---|---|---|---|---|---|---|---|---|
| **$CT_{max}$** | 738 | **0.085** | **2.84** | **0.025** | **0.146** | -713.6 | 0.971 | 0.030 | 0.175 | 0.198 | 0.567 | <0.001 | 0.416 |
| **$CT_{min}$** | 556 | **0.154** | **7.18** | **0.111** | **0.197** | -276.6 | 0.992 | 0.363 | <0.001 | <0.001 | 0.629 | <0.001 | 0.366 |

**Supplementary Table 14** Intercept multi-level meta-analytic, random effects models for upper and lower thermal limit, $CT_{max}$ and $CT_{min}$ respectively, without Drosophilidae data. CI.lb: lower bound of the 95% confidence interval; CI.ub: upper bound of the 95% confidence interval. $R^2_{marg.}$: $R^2$ marginal, the variance explained only by moderators. $R^2_{cond.}$: $R^2$ conditional, the variance explained by moderators and random effects. Significant results (95% CIs do not span 0) are highlighted in bold.

| **Limit** | **k** | **estimate** | **t** | **CI.lb** | **CI.ub** | **AICc** | **$I^2_{total}$** | **$I^2_{study}$** | **$I^2_{phylogeny}$** | **$I^2_{species}$** | **$I^2_{row}$** | **$R^2_{marg.}$** | **$R^2_{cond.}$** |
|---|---|---|---|---|---|---|---|---|---|---|---|---|---|
| **$CT_{max}$** | 421 | **0.094** | **2.77** | **0.026** | **0.162** | -267.4 | 0.978 | 0.427 | 0.132 | 0.205 | 0.314 | <0.001 | 0.679 |
| **$CT_{min}$** | 377 | **0.121** | **4.73** | **0.070** | **0.173** | -339.6 | 0.981 | <0.001 | 0.088 | 0.178 | 0.715 | <0.001 | 0.27 |

**Supplementary Table 15** Intercept multi-level meta-analytic, random effects models for upper and lower thermal limit, $CT_{max}$ and $CT_{min}$ respectively, with Drosophilidae data only. CI.lb: lower bound of the 95% confidence interval; CI.ub: upper bound of the 95% confidence interval. $R^2_{marg.}$: $R^2$ marginal, the variance explained only by moderators. $R^2_{cond.}$: $R^2$ conditional, the variance explained by moderators and random effects. Significant results (95% CIs do not span 0) are highlighted in bold.

| **Limit** | **k** | **estimate** | **t** | **CI.lb** | **CI.ub** | **AICc** | **$I^2_{total}$** | **$I^2_{study}$** | **$I^2_{phylogeny}$** | **$I^2_{species}$** | **$I^2_{row}$** | **$R^2_{marg.}$** | **$R^2_{cond.}$** |
|---|---|---|---|---|---|---|---|---|---|---|---|---|---|
| **$CT_{max}$** | 382 | 0.036 | 0.45 | -0.140 | 0.212 | -533.2 | 0.971 | 0.014 | 0.325 | 0.111 | 0.521 | <0.001 | 0.463 |
| **$CT_{min}$** | 194 | **0.225** | **3.20** | **0.066** | **0.383** | -14.3 | 0.997 | 0.477 | <0.001 | <0.001 | 0.520 | <0.001 | 0.479 |

**Supplementary Table 16** Univariate multi-level meta-analytic, random effects models for upper critical thermal limit ($CT_{max}$) ARR (Acclimation Response Ratio), with Drosophilidae data only. Significant results (95% CIs do not overlap between groups or significant regressions for continuous variables) are highlighted in bold. CI.lb: lower bound of the 95% confidence interval; CI.ub: upper bound of the 95% confidence interval. $R^2_{marg.}$: $R^2$ marginal, the variance explained only by moderators. $R^2_{cond.}$: $R^2$ conditional, the variance explained by moderators and random effects.

| Model | Comparison | k | estimate | t | CI.lb | CI.ub | AICc | $I^2_{total}$ | $I^2_{study}$ | $I^2_{phylogeny}$ | $I^2_{species}$ | $I^2_{row}$ | $R^2_{marg.}$ | $R^2_{cond.}$ |
|---|---|---|---|---|---|---|---|---|---|---|---|---|---|---|
| ***~duration*** | | 149 | **<-0.001** | **-2.03** | **<-0.001** | **<-0.001** | -413.5 | 0.779 | <-0.001 | 0.115 | 0.115 | 0.549 | 0.053 | 0.333 |
| | Intercept | - | 0.044 | 4.97 | 0.023 | 0.066 | - | - | - | - | - | - | - | - |
| *~ramp rate* | | 382 | 0.002 | 0.02 | -0.245 | 0.249 | -528.7 | 0.959 | 0.018 | 0.104 | 0.104 | 0.732 | <0.001 | 0.237 |
| | Intercept | - | 0.042 | 1.97 | -0.006 | 0.091 | - | - | - | - | - | - | - | - |
| *~mass* | | 351 | -0.003 | -0.19 | -0.038 | 0.032 | -461.3 | 0.964 | 0.016 | 0.122 | 0.122 | 0.703 | <0.001 | 0.270 |
| | Intercept | - | 0.047 | 1.49 | -0.025 | 0.119 | - | - | - | - | - | - | - | - |
| *~acclimation stage* | Early life | 382 | 0.052 | 2.24 | 0.006 | 0.098 | -528.5 | 0.959 | 0.009 | 0.112 | 0.112 | 0.726 | 0.011 | 0.251 |
| | Adulthood | - | 0.025 | 1.32 | -0.012 | 0.062 | - | - | - | - | - | - | - | - |
| | Whole life | - | 0.051 | 2.92 | 0.017 | 0.085 | - | - | - | - | - | - | - | - |
| *~endpoint* | Activity | 382 | 0.032 | 0.032 | -0.012 | 0.075 | -527.5 | 0.959 | 0.013 | 0.110 | 0.110 | 0.726 | 0.008 | 0.249 |
| | Stimulus response | - | 0.056 | 0.056 | 0.009 | 0.103 | - | - | - | - | - | - | - | - |
| | Motor response | - | 0.047 | 0.047 | -0.05 | 0.140 | - | - | - | - | - | - | - | - |
| *~latitude* | | 380 | <0.001 | -0.21 | -0.001 | 0.001 | -524.1 | 0.959 | 0.029 | 0.102 | 0.102 | 0.727 | <0.001 | 0.242 |
| | Intercept | - | 0.046 | 1.79 | -0.013 | 0.106 | - | - | - | - | - | - | - | - |
| ***~sex*** | **Female** | 382 | **0.028** | **1.59** | **-0.007** | **0.063** | -533.3 | 0.959 | 0.008 | 0.111 | 0.111 | 0.728 | 0.017 | 0.253 |
| | **Male** | - | **0.060** | **3.29** | **0.024** | **0.096** | - | - | - | - | - | - | - | - |

**Supplementary Table 17** Univariate multi-level meta-analytic, random effects models for lower critical thermal limit ($CT_{min}$) ARR (Acclimation Response Ratio), with Drosophilidae data only. Significant results (95% CIs do not overlap between groups or significant regressions for continuous variables) are highlighted in bold. CI.lb: lower bound of the 95% confidence interval; CI.ub: upper bound of the 95% confidence interval. $R^2_{marg.}$: $R^2$ marginal, the variance explained only by moderators. $R^2_{cond.}$: $R^2$ conditional, the variance explained by moderators and random effects.

| Model | Comparison | k | estimate | t | CI.lb | CI.ub | AICc | $I^2_{total}$ | $I^2_{study}$ | $I^2_{phylogeny}$ | $I^2_{species}$ | $I^2_{row}$ | $R^2_{marg.}$ | $R^2_{cond.}$ |
|---|---|---|---|---|---|---|---|---|---|---|---|---|---|---|
| ***~duration*** | | 41 | **-0.001** | **-5.70** | **-0.002** | **-0.001** | -10.9 | 0. 997 | 0.088 | <0.001 | <0.001 | 0.091 | 0.469 | 0.516 |
| | Intercept | - | 0.171 | 3.61 | -0.431 | 0.773 | - | - | - | - | - | - | - | - |
| *~ramp rate* | | 194 | 0.011 | 0.04 | -0.500 | 0.523 | -13.0 | 0. 997 | 0.494 | <0.001 | <0.001 | 0.502 | <0.001 | 0.496 |
| | Intercept | - | 0.222 | 2.24 | -0.007 | 0.450 | - | - | - | - | - | - | - | - |
| *~mass* | | 184 | -0.005 | -0.23 | -0.056 | 0.045 | -3.6 | 0. 997 | 0.473 | <0.001 | <0.001 | 0.524 | <0.001 | 0.475 |
| | Intercept | - | 0.233 | 3.08 | 0.058 | 0.409 | - | - | - | - | - | - | - | - |
| *~acclimation stage* | Early life | 194 | 0.356 | 3.78 | 0.170 | 0.543 | -20.3 | 0.997 | 0.595 | <0.001 | <0.001 | 0.402 | 0.077 | 0.628 |
| | Adulthood | - | 0.241 | 2.65 | 0.061 | 0.421 | - | - | - | - | - | - | - | - |
| | Whole life | - | 0.121 | 1.32 | -0.060 | 0.302 | - | - | - | - | - | - | - | - |
| | Activity | 194 | 0.149 | 1.07 | -0.208 | 0.506 | -13.9 | 0.997 | 0.529 | <0.001 | <0.001 | 0.468 | 0.107 | 0.581 |
| | Clinging | - | 0.084 | 0.36 | -0.507 | 0.675 | - | - | - | - | - | - | - | - |
| | Righting | - | 0.333 | 1.80 | -0.144 | 0.809 | - | - | - | - | - | - | - | - |
| | Stimulus response | - | 0.343 | 2.48 | -0.012 | 0.699 | - | - | - | - | - | - | - | - |
| | Motor response | - | 0.072 | 0.30 | -0.547 | 0.691 | - | - | - | - | - | - | - | - |
| *~latitude* | | 191 | -0.001 | -0.36 | -0.003 | 0.002 | -10.4 | 0.997 | 0.502 | <0.001 | <0.001 | 0.495 | 0.001 | 0.503 |
| | Intercept | - | 0.237 | 2.41 | -0.004 | 0.475 | - | - | - | - | - | - | - | - |
| *~sex* | Female | 194 | 0.229 | 2.77 | 0.066 | 0.392 | -11.9 | 0.997 | 0.500 | <0.001 | <0.001 | 0.497 | 0.009 | 0.506 |
| | Male | - | 0.197 | 2.21 | 0.021 | 0.372 | - | - | - | - | - | - | - | - |
| | Mixed | - | 0.293 | 1.35 | -0.222 | 0.809 | - | - | - | - | - | - | - | - |

**Publication bias**

**Supplementary Table 18** Egger's regression test (two-sided) for intercept multi-level, random effects meta-analytic model for upper critical thermal limit ($CT_{max}$) ARR (Acclimation Response Ratio). SE: standard error. CI.lb: lower bound of the 95% confidence interval; CI.ub: upper bound of the 95% confidence interval. Significant results are highlighted in bold.

| **Moderator** | **k** | **estimate** | **t** | **SE** | **CI.lb** | **CI.ub** |
|---|---|---|---|---|---|---|
| Intercept | 803 | 0.073 | 2.37 | 0.031 | 0.011 | 0.134 |
| **√variance** | - | **0.288** | 2.17 | 0.133 | **0.028** | **0.548** |

**Supplementary Table 19** Egger's regression test (two-sided) was significant for intercept multi-level, random effects meta-analytic model for upper critical thermal limit ($CT_{max}$) ARR (Acclimation Response Ratio) so $SE^2$ was run as a moderator to find predicted estimate without publication bias. SE: standard error. CI.lb: lower bound of the 95% confidence interval; CI.ub: upper bound of the 95% confidence interval. Significant results are highlighted in bold.

| **Moderator** | **k** | **estimate** | **t** | **SE** | **CI.lb** | **CI.ub** |
|---|---|---|---|---|---|---|
| Intercept | 803 | 0.091 | 2.97 | 0.031 | 0.030 | 0.152 |
| **$SE^2$** | - | **0.043** | 0.67 | 0.06v5 | **-0.084** | **0.170** |

**Supplementary Table 20** Egger's regression test (two-sided) for the best multi-level, random effects meta-analytic model (as selected using the MuMIn package) for upper critical thermal limit ($CT_{max}$) ARR (Acclimation Response Ratio). SE: standard error. CI.lb: lower bound of the 95% confidence interval; CI.ub: upper bound of the 95% confidence interval. Significant results are highlighted in bold.

| **Moderator** | **k** | **estimate** | **t** | **SE** | **CI.lb** | **CI.ub** |
|---|---|---|---|---|---|---|
| Intercept | 803 | 0.116 | 3.43 | 0.034 | 0.048 | 0.183 |
| **√variance** | - | **0.288** | 2.17 | 0.133 | **0.028** | **0.548** |
| **Development: Homogeneous** | - | **-0.088** | -2.14 | 0.041 | **-0.169** | **-0.006** |

**Supplementary Table 21** Egger's regression test (two-sided) for intercept multi-level, random effects meta-analytic model for lower critical thermal limit ($CT_{min}$) ARR (Acclimation Response Ratio). SE: standard error. CI.lb: lower bound of the 95% confidence interval; CI.ub: upper bound of the 95% confidence interval. Significant results are highlighted in bold.

| **Moderator** | **k** | **estimate** | **t** | **SE** | **CI.lb** | **CI.ub** |
|---|---|---|---|---|---|---|
| Intercept | 571 | 0.116 | 4.67 | 0.025 | 0.066 | 0.165 |
| **√variance** | - | **0.621** | 2.21 | 0.282 | **0.068** | **1.174** |

**Supplementary Table 22** Egger's regression test (two-sided) was significant for intercept multi-level, random effects meta-analytic model for lower critical thermal limit ($CT_{min}$) ARR (Acclimation Response Ratio) so $SE^2$ was run as a moderator to find predicted estimate without publication bias. SE: standard error. CI.lb: lower bound of the 95% confidence interval; CI.ub: upper bound of the 95% confidence interval. Significant results are highlighted in bold.

| **Moderator** | **k** | **estimate** | **t** | **SE** | **CI.lb** | **CI.ub** |
|---|---|---|---|---|---|---|
| Intercept | 571 | 0.144 | 6.94 | 0.021 | 0.102 | 0.185 |
| **$SE^2$** | - | **0.635** | 1.28 | 0.497 | **-0.341** | **1.611** |

**Supplementary Table 23** Egger's regression test (two-sided) for the best multi-level, random effects meta-analytic model (as selected using the MuMIn package) for lower critical thermal limit ($CT_{min}$) ARR (Acclimation Response Ratio). SE: standard error. CI.lb: lower bound of the 95% confidence interval; CI.ub: upper bound of the 95% confidence interval. Significant results are highlighted in bold.

| **Moderator** | **k** | **estimate** | **t** | **SE** | **CI.lb** | **CI.ub** |
|---|---|---|---|---|---|---|
| Intercept | 571 | 0.068 | 1.92 | 0.035 | -0.003 | 0.139 |
| **√variance** | - | **0.695** | 2.46 | 0.283 | **0.140** | **1.249** |
| Source: laboratory | - | 0.064 | 1.85 | 0.035 | -0.004 | 0.133 |

**Supplementary Table 24** Univariate multi-level meta-analytic, random effects models for upper critical thermal limit ($CT_{max}$) ARR (Acclimation Response Ratio), investigating year as a moderator. SE: standard error. CI.lb: lower bound of the 95% confidence interval; CI.ub: upper bound of the 95% confidence interval. Significant results are highlighted in bold.

| **Moderator** | **k** | **estimate** | **t** | **SE** | **CI.lb** | **CI.ub** |
|---|---|---|---|---|---|---|
| Intercept | 803 | 1.73 | 0.42 | 4.157 | -6.59 | 10.046 |
| Year | - | -0.001 | -0.39 | 0.002 | -0.005 | 0.003 |

**Supplementary Table 25** Univariate multi-level meta-analytic, random effects models for lower critical thermal limit ($CT_{min}$) ARR (Acclimation Response Ratio), investigating year as a moderator. SE: standard error. CI.lb: lower bound of the 95% confidence interval; CI.ub: upper bound of the 95% confidence interval. Significant results are highlighted in bold.

| **Moderator** | **k** | **estimate** | **t** | **SE** | **CI.lb** | **CI.ub** |
|---|---|---|---|---|---|---|
| Intercept | 571 | 4.103 | 0.60 | 6.809 | -9.574 | 17.780 |
| Year | - | -0.002 | -0.58 | 0.003 | -0.009 | 0.005 |
