# Supplementary material for: Meta-analysis reveals weak but pervasive plasticity in insect thermal limits
Source: Nat Commun. 2022 Sep 8;13:5292. doi: 10.1038/s41467-022-32953-2 (PMC9458737; doi:10.1038/s41467-022-32953-2)
Supplement: Supplementary file 3 — Description of Additional Supplementary Information [file 41467_2022_32953_MOESM3_ESM.docx]

**Description of Additional Supplementary Files**

**File name**: Supplementary Data 1.

**Description**: Raw and processed (ARR, variance, precision) dataset for systematic meta-analysis of experimental studies on the plasticity of insects’ upper and lower critical thermal limits, including taxon-specific moderators to investigate variation in plasticity.

**File name**: Supplementary Data 2.

**Description**: List of the 74 references from which data (arithmetic mean, standard deviation (SD), sample size (N)) were extracted for the meta-analysis of experimental studies on the plasticity of insects’ upper and lower critical thermal limits.

**File name**: Supplementary Data 3.

**Description**: List of the 4 references excluded from the meta-analysis due to studies measuring a very large number of insects at one time, meaning the sample size and therefore precision of the study was inflated.

**File name**: Supplementary Data 4.

**Description**: List of the 40 references from which wet body mass data was extracted (if not included in the original study).
