# Supplementary material for: Meta-analysis reveals weak but pervasive plasticity in insect thermal limits
Source: Nat Commun. 2022 Sep 8;13:5292. doi: 10.1038/s41467-022-32953-2 (PMC9458737; doi:10.1038/s41467-022-32953-2)
Supplement: Supplementary file 8 — Reporting Summary [file 41467_2022_32953_MOESM8_ESM.pdf]

nature portfolio

Corresponding author(s): Hester Weaving

Last updated by author(s): Aug 17, 2022

Reporting Summary

Nature Portfolio wishes to improve the reproducibility of the work that we publish. This form provides structure for consistency and transparency in reporting. For further information on Nature Portfolio policies, see our Editorial Policies and the Editorial Policy Checklist.

Statistics

For all statistical analyses, confirm that the following items are present in the figure legend, table legend, main text, or Methods section.

n/a	Confirmed	
☐	☒	The exact sample size (*n*) for each experimental group/condition, given as a discrete number and unit of measurement
☐	☒	A statement on whether measurements were taken from distinct samples or whether the same sample was measured repeatedly
☐	☒	The statistical test(s) used AND whether they are one- or two-sided<br>*Only common tests should be described solely by name; describe more complex techniques in the Methods section.*
☐	☒	A description of all covariates tested
☐	☒	A description of any assumptions or corrections, such as tests of normality and adjustment for multiple comparisons
☐	☒	A full description of the statistical parameters including central tendency (e.g. means) or other basic estimates (e.g. regression coefficient) AND variation (e.g. standard deviation) or associated estimates of uncertainty (e.g. confidence intervals)
☐	☒	For null hypothesis testing, the test statistic (e.g. *F*, *t*, *r*) with confidence intervals, effect sizes, degrees of freedom and *P* value noted<br>*Give P values as exact values whenever suitable.*
☒	☐	For Bayesian analysis, information on the choice of priors and Markov chain Monte Carlo settings
☒	☐	For hierarchical and complex designs, identification of the appropriate level for tests and full reporting of outcomes
☐	☒	Estimates of effect sizes (e.g. Cohen's *d*, Pearson's *r*), indicating how they were calculated

Our web collection on statistics for biologists contains articles on many of the points above.

Software and code

Policy information about availability of computer code

Data collection	Data were extracted from peer-reviewed literature from Web of Science and Scopus published between January 1990 and November 2020 on the plasticity of insect critical thermal limits. Data were digitised from Figures using R package 'metaDigitise' (Version 1.0.1). Otherwise, data were extracted by HW from text, table or by contacting the corresponding author. The data used in this study are available on the OSF database under accession code: https://osf.io/cbhv4/
Data analysis	All analyses were completed in R version 4.0.3. Phylogenetic trees were constructed in the Open Tree of Life and R packages 'rotl' (Version 3.0.11) and 'ape' (Version 5.5). R package 'metafor' (Version 3.0-2) was used to perform multi-level, random effects models. We used the 'Dredge' function from the MuMin package (Version 1.43.17) to assess which combination of models had the best fit. Figures 1-4 were constructed using the orchaRd package (Version 0.0.0.9). The code used in this study are available on the OSF database under accession code: https://osf.io/cbhv4/

For manuscripts utilizing custom algorithms or software that are central to the research but not yet described in published literature, software must be made available to editors and reviewers. We strongly encourage code deposition in a community repository (e.g. GitHub). See the Nature Portfolio guidelines for submitting code & software for further information.

Data

Policy information about availability of data

All manuscripts must include a data availability statement. This statement should provide the following information, where applicable:

- Accession codes, unique identifiers, or web links for publicly available datasets
- A description of any restrictions on data availability
- For clinical datasets or third party data, please ensure that the statement adheres to our policy

The data used in this study are available on the OSF database under accession code: https://osf.io/cbhv4/. We provide raw and processed data. We also provide these data in the Supplementary Files as Supplementary Data 1

Field-specific reporting

Please select the one below that is the best fit for your research. If you are not sure, read the appropriate sections before making your selection.

☐ Life sciences ☐ Behavioural & social sciences ☒ Ecological, evolutionary & environmental sciences

For a reference copy of the document with all sections, see nature.com/documents/nr-reporting-summary-flat.pdf

Ecological, evolutionary & environmental sciences study design

All studies must disclose on these points even when the disclosure is negative.

Study description	We undertake a systematic meta-analysis of experimental studies on the plasticity of insects' upper and lower critical thermal limits, including taxon-specific moderators to investigate variation in plasticity.
Research sample	Data represent global studies that measure insect critical thermal limit plasticity. Peer-reviewed studies were selected for data extraction if they described dynamic tolerance assays where CTmax or CTmin was measured by ramping the temperature until a specified end point. We chose to only evaluate dynamic studies as it was a common metric used in thermal tolerance assays, removed additional sources of methodological heterogeneity, and was a metric already synthesised in other meta-analyses. We required studies to have at least two temperature treatments (including studies where a single treatment was compared to a control), perform a temperature acclimation treatment (all durations of acclimation, including acute hardening and longer-term chronic acclimation, were included and fluctuating temperatures were allowed), and be undertaken in a laboratory. Studies were not included if any variables in addition to temperature were modified (excluding named moderators). Studies were also excluded if the endpoint was recorded for a proportion of the insects assayed only (e.g. CTmax80). A reference list for studies used in the analysis are provided in the Supplementary Information.
Sampling strategy	Each step was reported according to the PRISMA (Preferred Reporting Items for Systematic Reviews and Meta-Analyses) guidelines. Sample size was not predetermined as data were extracted from all studies that met our search criteria. Sample size was checked for all groups and analyses were not run if sample sizes were too small.
Data collection	In total, Web of Science and Scopus databases found 12,139 unique results. Study abstracts were scanned manually for suitability by a single observer (HW) and selected studies were further examined by their methodology. Data were extracted (arithmetic mean, standard deviation (SD), sample size (N)) from 60 and 52 articles, comprising 92 and 74 species, for CTmax and CTmin respectively. Data were extracted from Figures, Tables and text or the author was contacted to obtain data. Data were recorded in Excel. Moderators were extracted either from the study itself or from published studies and meta-analyses. References for studies from which data were extracted can be found in the Supplementary Information in Supplementary Data 2-4.
Timing and spatial scale	Searches were performed in Web of Science and Scopus between July and November 2020 by HW. Searches included globally published studies but were limited to those published in English and were biased towards Europe and North America. The search only included studies published between January 1990 and November 2020. Papers were excluded if they were published before 1990 as Web of Science only had 3 hits pre 1990 so if included, may have introduced bias between the databases. Additionally, methodologies from earlier papers differed drastically eg measuring % individuals falling from a column, which was not easily comparable to other data collected.
Data exclusions	Four studies (cited in the Supplementary Data 3) where a very large number of insects were measured were removed from the CTmax dataset as the unusually large sample sizes (n > 700) grossly inflated the study weight and it was deemed this number of insects could not be accurately assessed in one critical thermal limit assay.
Reproducibility	Each step was reported according to the PRISMA (Preferred Reporting Items for Systematic Reviews and Meta-Analyses) guidelines. Methodology of data collection and analysis are presented to enhance reproducibility the data. Code and data have been made available so that the study can be reproduced on OSF at: https://osf.io/cbhv4/
Randomization	Not relevant to a meta-analysis study.
Blinding	Blinding was not relevant to this study. Data were included or excluded by one author (HW) based on a list of criteria.

Did the study involve field work? ☐ Yes ☒ No

Reporting for specific materials, systems and methods

We require information from authors about some types of materials, experimental systems and methods used in many studies. Here, indicate whether each material, system or method listed is relevant to your study. If you are not sure if a list item applies to your research, read the appropriate section before selecting a response.

Materials & experimental systems

n/a	Involved in the study
☒	☐ Antibodies
☒	☐ Eukaryotic cell lines
☒	☐ Palaeontology and archaeology
☒	☐ Animals and other organisms
☒	☐ Human research participants
☒	☐ Clinical data
☒	☐ Dual use research of concern

Methods

n/a	Involved in the study
☒	☐ ChIP-seq
☒	☐ Flow cytometry
☒	☐ MRI-based neuroimaging
